# Racial and Ethnic Disparities in Receipt of Behavioral Treatment Among Children With Autism Spectrum Disorder in the United States

**DOI:** 10.7759/cureus.116117

**Published:** 2026-09-11

**Authors:** Andrew Han, Narae Lee

**Affiliations:** 1 Psychology, Diamond Bar High School, Diamond Bar, USA; 2 Psychology, Department of State Hospitals - Metropolitan, Norwalk, USA

**Keywords:** behavior therapy, children with autism spectrum disorders (asd), minority health, national survey of children's health, racial disparity

## Abstract

Background and objective

Minority children have delayed diagnosis of autism spectrum disorder (ASD). However, it is unclear whether racial disparities exist in minority children receiving treatment even after a diagnosis, which may further negatively affect their cognitive, social, and behavioral development. We examined caregiver-reported receipt of behavioral treatment across racial and ethnic groups to determine whether minority children younger than five years with ASD were less likely to receive treatment than White children in the United States.

Methods

From the 2021-2022 National Survey of Children’s Health, we included children with a diagnosis of ASD who were younger than five years of age. We used self-reported responses from parents or caregivers on whether their children had received behavioral treatment for ASD during the previous 12 months. We compared behavioral treatment for ASD by racial and ethnic groups by conducting chi-square tests. We performed multivariable logistic regression models to determine the association between racial and ethnic groups and behavioral treatment after adjustment for age, sex, severity of ASD, parent education, and Medicaid status.

Results

Among 790 children aged ≤5 years (51 (6.5%) Asian, 143 (18.1%) Hispanic, 78 (9.9%) non-Hispanic (NH) Black, 434 (54.9%) NH White, 84 (10.6%) multi-race; 615 (77.9%) male), 564 (71.4%) received behavioral treatment. Multi-race children had the highest percentage of behavioral treatment (n = 65, 77.4%), followed by Hispanic (n = 106, 74.1%), NH White (n = 314, 72.4%), NH Asian (n = 35, 68.6%), and NH Black (n = 44, 56.4%) children (*p* = 0.027). Compared to NH White children, NH Black children were less likely to be treated (adjusted OR (95% CI) = 0.47 (0.28, 0.79)). The odds of treatment for Asian, Hispanic, and multiracial children compared to NH White children were not statistically significant.

Conclusions

NH Black children with ASD were significantly less likely than NH White children to have caregiver-reported receipt of behavioral treatment. These findings underscore the importance of continued efforts to promote equitable receipt of behavioral treatment among children with ASD, particularly for NH Black children.

## Introduction

Autism spectrum disorder (ASD) is a neurodevelopmental condition that can affect children’s social, communication, and behavioral development [1]. The increasing diagnosis of ASD in young children (one in 31) necessitates timely diagnosis and treatment [2,3]. Historically, minority children have been impacted by delayed diagnosis of ASD compared to White children [4-6]. While recent studies have shown that gaps in the age of ASD diagnosis among racial groups have narrowed [7], racial disparities in access to behavioral treatment following diagnosis remain insufficiently explored.

The standard treatment for ASD includes behavioral therapy, which benefits children the earlier it is applied [8]. If racial and ethnic minority children face disparities in receiving behavioral treatment even after diagnosis, this could negatively affect their cognitive, social, and behavioral development. Few studies have specifically examined whether racial and ethnic differences exist in caregiver-reported receipt of behavioral treatment among young children with ASD after diagnosis. The National Survey of Children’s Health (NSCH) provides a large national sample of children with ASD and an opportunity to evaluate caregiver-reported receipt of behavioral treatment across racial and ethnic groups [9]. In this study, we examined receipt of behavioral therapy across racial and ethnic groups using the 2021-2022 NSCH. We hypothesized that non-Hispanic (NH) Asian, Black, multiracial, and Hispanic children with ASD would be less likely to receive treatment for ASD compared to White children.

This article was previously presented as an abstract at the 2024 American Academy of Pediatric Neuropsychology (AAPdN) 10th Annual Conference on April 19-21, 2024.

## Materials and methods

We conducted a cross-sectional data analysis of the NSCH from 2021 to 2022. The NSCH is a nationally representative annual survey of children from birth to age 17, designed to provide national and state-level estimates of children’s physical and emotional health in the United States (US) [9]. All NSCH data are reported by parents or guardians. The NSCH is sponsored and managed by the Maternal and Child Health Bureau of the Health Resources and Services Administration and administered by the US Census Bureau. The NSCH uses an address-based sampling design in which households are stratified within each state by their likelihood of containing children, with one child randomly selected per household as the survey subject. As this study used publicly available, de-identified data, institutional review board approval was not required.

We analyzed children younger than five years whose parent or caregiver reported that a health care provider had diagnosed the child with ASD and that the child currently had the condition based on NSCH items (K2Q35A and K2Q35B). We chose this age restriction because early identification and initiation of behavioral treatment are critical during this developmental window, consistent with the CDC recommendations for ASD screening at 18-24 months and evidence supporting early intervention in the first few years of life [10]. We restricted our sample to individuals who responded to the autism treatment question: “At any time during the past 12 months, did this child receive behavioral treatment for autism, ASD, Asperger’s disorder, or pervasive developmental disorder, such as training or an intervention that you or this child received to help with their behavior?” Anyone who did not respond to the question was excluded (n = 4). The outcome of interest in this study was ASD behavioral treatment. The exposure of interest was the race and ethnicity of children with a current diagnosis of ASD. Race and ethnicity were categorized as NH Asian, NH Black, NH White, multiracial, and Hispanic. Missing race and Hispanic origin data in the NSCH were imputed using hot-deck imputation [11]. We collected information on children and their parents/guardians, including child age, child age at ASD diagnosis, child sex, age of mother, parent education (less than high school, high school, some college or associate degree, college degree, or higher), child’s ASD severity (mild, moderate, or severe), and Medicaid coverage. Of these, child age, sex, ASD severity, parent education, and Medicaid coverage were selected a priori for the adjusted regression model based on prior literature demonstrating their associations with ASD diagnosis, severity, and access to care, and their potential role as confounders [4,5,12,13]. Because this study was conducted to examine the association between race/ethnicity and behavioral treatment rather than to generate national prevalence estimates, analyses were conducted using unweighted data and did not incorporate the NSCH complex survey design. Sampling weights were not applied.

We described the study population using mean and SD for continuous variables and frequency and percentages for categorical variables. We compared population characteristics and behavioral treatment for ASD across racial and ethnic groups using chi-square tests for categorical variables. For continuous variables, we used one-way ANOVA and post hoc pairwise comparisons using Tukey’s method. We conducted univariate logistic regression models to determine the association between racial and ethnic groups and behavioral therapy. For the multivariable analysis, 25 observations (3.2%) with missing covariate data were excluded (ASD severity, n = 5; Medicaid coverage, n = 21), resulting in a final analytic sample of 765 children.

We presented crude ORs and adjusted ORs with 95% CIs using NH White as a reference group. A p-value of less than 0.05 was considered statistically significant. All analyses were conducted using SAS 9.4 (SAS Institute, Cary, NC, US) and RStudio 2021.09.1 on Posit Cloud (Posit, Boston, MA, US).

## Results

We included 790 children under the age of five years. The mean ± SD age of the children was 3.82 ± 1.08 years, and 615 (77.9%) children were male. Race and ethnicity distributions were 51 (6.5%) NH Asian, 143 (18.1%) Hispanic, 78 (9.9%) NH Black, 434 (54.9%) NH White, and 84 (10.6%) multiracial. Among the total, 308 (39.2%), 364 (46.4%), and 113 (14.4%) children had mild, moderate, and severe ASD, respectively (Table 1).

**Table 1:** Characteristics of children with ASD stratified by race and ethnicity. Data are presented as mean ± SD and n (%). ^*^ Chi-square statistics for categorical variables and F-statistics from ANOVA tests for continuous variables. ^†^ Percentages are calculated using non-missing observations for each variable. ASD severity data were missing for 5 children (0.6%), and Medicaid coverage data were missing for 21 children (2.7%). ASD, autism spectrum disorder; NH, non-Hispanic

Variable	Total (n = 790)	Asian (n = 51)	NH Black (n = 78)	Hispanic (n = 143)	Multiracial (n = 84)	NH White (n = 434)	Test statistics^*^	p-value
Age	3.82 ± 1.08	3.53 ± 1.03	3.78 ± 1.01	3.66 ± 1.20	3.79 ± 0.96	3.92 ± 1.07	2.811	0.025
Age at diagnosis	2.54 ± 0.94	2.23 ± 0.87	2.69 ± 1.00	2.36 ± 0.89	2.56 ± 0.87	2.61 ± 0.95	3.611	0.006
Sex								
Male	615 (77.9%)	41 (80.4%)	61 (78.2%)	108 (75.5%)	71 (84.5%)	334 (77.0%)	3.015	0.555
Female	175 (22.2%)	10 (19.6%)	17 (21.8%)	35 (24.5%)	13 (15.5%)	100 (23.0%)		
Age of mother (at birth)	30.47 ± 5.95	31.80 ± 5.33	31.59 ± 5.47	29.81 ± 6.36	31.95 ± 5.83	30.05 ± 5.91	3.559	0.007
Parent education								
Less than high school or high school (including vocational, trade, or business school)	184 (23.3%)	3 (5.9%)	20 (25.6%)	46 (32.2%)	17 (20.2%)	98 (22.6%)	29.234	<0.001
Some college or an associate’s degree	216 (27.3%)	9 (17.7%)	19 (24.4%)	39 (27.3%)	17 (20.2%)	132 (30.4%)		
College degree or higher	390 (49.4%)	39 (76.5%)	39 (50.0%)	58 (40.6%)	50 (59.5%)	204 (47.0%)		
ASD severity^†^								
Mild	308 (39.2%)	20 (39.2%)	35 (44.9%)	55 (39.0%)	35 (41.7%)	163 (37.8%)	4.637	0.796
Moderate	364 (46.4%)	26 (51.0%)	30 (38.5%)	67 (47.5%)	40 (47.6%)	201 (46.6%)		
Severe	113 (14.4%)	5 (10.0%)	13 (16.7%)	19 (13.5%)	9 (10.7%)	67 (15.5%)		
Medicaid coverage^†^	440 (57.2%)	13 (28.9%)	55 (71.4%)	90 (65.2%)	47 (56.6%)	235 (55.6%)	28.255	<0.001

The mean ± SD age at ASD diagnosis was 2.54 ± 0.94 years. NH Black children had the highest mean age at diagnosis (Table 1); however, pairwise comparisons were not statistically significant (Table 2). The percentages of ASD severity were similar across racial and ethnic groups (p = 0.796). Hispanic and NH Black parents of children with ASD had the two highest percentages of less than a high school or high school education (32.2% and 25.6%, respectively) compared with other racial and ethnic groups. NH Black and Hispanic families of children with ASD had the highest percentages of Medicaid coverage, 71.4% and 65.2%, respectively.

**Table 2:** Post hoc pairwise comparisons of continuous characteristics by race and ethnicity. Mean difference is the difference in means between the two groups (Group 2 minus Group 1). p-values were adjusted using Tukey’s method for multiple pairwise comparisons. NH, non-Hispanic

Characteristic	Group 1	Group 2	Mean difference	95% CI	Tukey-adjusted p-value
Age	Asian	NH Black	0.253	(-0.277, 0.782)	0.688
	Asian	Hispanic	0.128	(-0.351, 0.607)	0.950
	Asian	Multi-race	0.256	(-0.265, 0.778)	0.664
	Asian	NH White	0.395	(-0.040, 0.830)	0.096
	NH Black	Hispanic	-0.125	(-0.538, 0.289)	0.923
	NH Black	Multi-race	0.004	(-0.458, 0.466)	1.000
	NH Black	NH White	0.142	(-0.219, 0.503)	0.820
	Hispanic	Multi-race	0.128	(-0.276, 0.532)	0.908
	Hispanic	NH White	0.267	(-0.017, 0.550)	0.076
	Multi-race	NH White	0.138	(-0.212, 0.489)	0.817
Age at diagnosis	Asian	NH Black	0.455	(-0.021, 0.932)	0.069
	Asian	Hispanic	0.122	(-0.312, 0.556)	0.939
	Asian	Multi-race	0.322	(-0.147, 0.790)	0.330
	Asian	NH White	0.377	(-0.016, 0.771)	0.067
	NH Black	Hispanic	-0.333	(-0.704, 0.038)	0.102
	NH Black	Multi-race	-0.134	(-0.544, 0.277)	0.901
	NH Black	NH White	-0.078	(-0.401, 0.245)	0.965
	Hispanic	Multi-race	0.199	(-0.161, 0.560)	0.554
	Hispanic	NH White	0.255	(-0.000, 0.511)	0.051
	Multi-race	NH White	0.056	(-0.255, 0.366)	0.988
Age of mother at birth	Asian	NH Black	-0.205	(-3.165, 2.755)	1.000
	Asian	Hispanic	-1.991	(-4.653, 0.670)	0.245
	Asian	Multi-race	0.151	(-2.750, 3.052)	1.000
	Asian	NH White	-1.748	(-4.166, 0.670)	0.278
	NH Black	Hispanic	-1.786	(-4.107, 0.535)	0.219
	NH Black	Multi-race	0.357	(-2.236, 2.949)	0.996
	NH Black	NH White	-1.543	(-3.580, 0.494)	0.234
	Hispanic	Multi-race	2.143	(-0.103, 4.388)	0.070
	Hispanic	NH White	0.243	(-1.328, 1.815)	0.993
	Multi-race	NH White	-1.899	(-3.850, 0.051)	0.061

Among the total children with ASD, 564 (71.4%) received behavioral therapy. Multiracial children had the highest percentage of behavioral treatment (77.4%), followed by Hispanic (74.1%), NH White (72.4%), NH Asian (68.6%), and NH Black (56.4%) children (p = 0.027) (Table 3).

**Table 3:** Percentage of children receiving behavioral therapy by race and ethnicity. ^*^ Chi-square test statistics NH, non-Hispanic

Received behavior therapy	Asian (n = 51)	NH Black (n = 78)	Hispanic (n = 143)	Multi-race (n = 84)	NH White (n = 434)	Test statistics^*^	p-value
Yes	35 (68.6%)	44 (56.4%)	106 (74.1%)	65 (77.4%)	314 (72.4%)	10.957	0.027
No	16 (31.4%)	34 (43.6%)	37 (25.9%)	19 (22.6%)	120 (27.6%)		

In the unadjusted analysis, NH Black race/ethnicity compared with NH White race/ethnicity was associated with decreased odds of behavioral treatment (OR (95% CI) = 0.49 (0.30, 0.81)) (Table 4). The odds of treatment for NH Asian, Hispanic, and multiracial children compared with NH White children were not statistically significant. In the adjusted analysis, we controlled for age, sex, ASD severity, parent education, and Medicaid coverage. Compared with NH White children, NH Black children had decreased odds of behavioral treatment (adjusted OR (95% CI) = 0.47 (0.28, 0.79)). In the adjusted analysis, the odds of treatment for the other racial and ethnic groups were not statistically significant.

**Table 4:** Crude and adjusted ORs (95% CI) of behavioral therapy by race and ethnicity. Logistic regression models were used to obtain crude and adjusted ORs. Dependent variable: self-reported ASD behavioral treatment receipt (yes to the question, “At any time during the past 12 months, did this child receive behavioral treatment for autism, ASD, Asperger’s disorder, or pervasive developmental disorder, such as training or an intervention that you or this child received to help with their behavior?”). Independent variable: race/ethnicity, with NH White as the reference category. Covariates for the adjusted OR: age, sex, ASD severity, parent education, and Medicaid coverage. Crude model: Final analytic sample, n = 790; likelihood ratio χ² = 10.37; pseudo R² = 0.019. Adjusted model: Final analytic sample, n = 765, after excluding observations with missing data (n = 25); likelihood ratio χ² = 63.485; pseudo R² = 0.115. NH, non-Hispanic

Variable	Asian	NH Black	Hispanic	Multi-race
Crude β (SE)	-0.179 (0.320)	-0.704 (0.252)	0.091 (0.219)	0.268 (0.282)
Crude OR	0.84 (0.45, 1.60)	0.49 (0.30, 0.81)	1.09 (0.72, 1.70)	1.31 (0.77, 2.32)
Crude p-values	0.576	0.005	0.679	0.342
Adjusted β (SE)	-0.337 (0.353)	-0.750 (0.265)	0.277 (0.238)	0.207 (0.294)
Adjusted OR	0.69 (0.35, 1.42)	0.47 (0.28, 0.79)	1.27 (0.80, 2.05)	1.23 (0.70, 2.24)
Adjusted p-values	0.299	0.004	0.312	0.481

Other factors independently associated with the odds of ASD behavioral treatment are shown in Figure 1. Children with mild ASD severity had lower odds of treatment than those with severe ASD (adjusted OR (95% CI) = 0.36 (0.21, 0.62)). Children with lower parent/guardian education also had lower odds of ASD behavioral treatment than those with higher education. Older age of children was modestly associated with the odds of ASD treatment, while sex and Medicaid coverage were not associated with the odds of ASD treatment.

**Figure 1:**
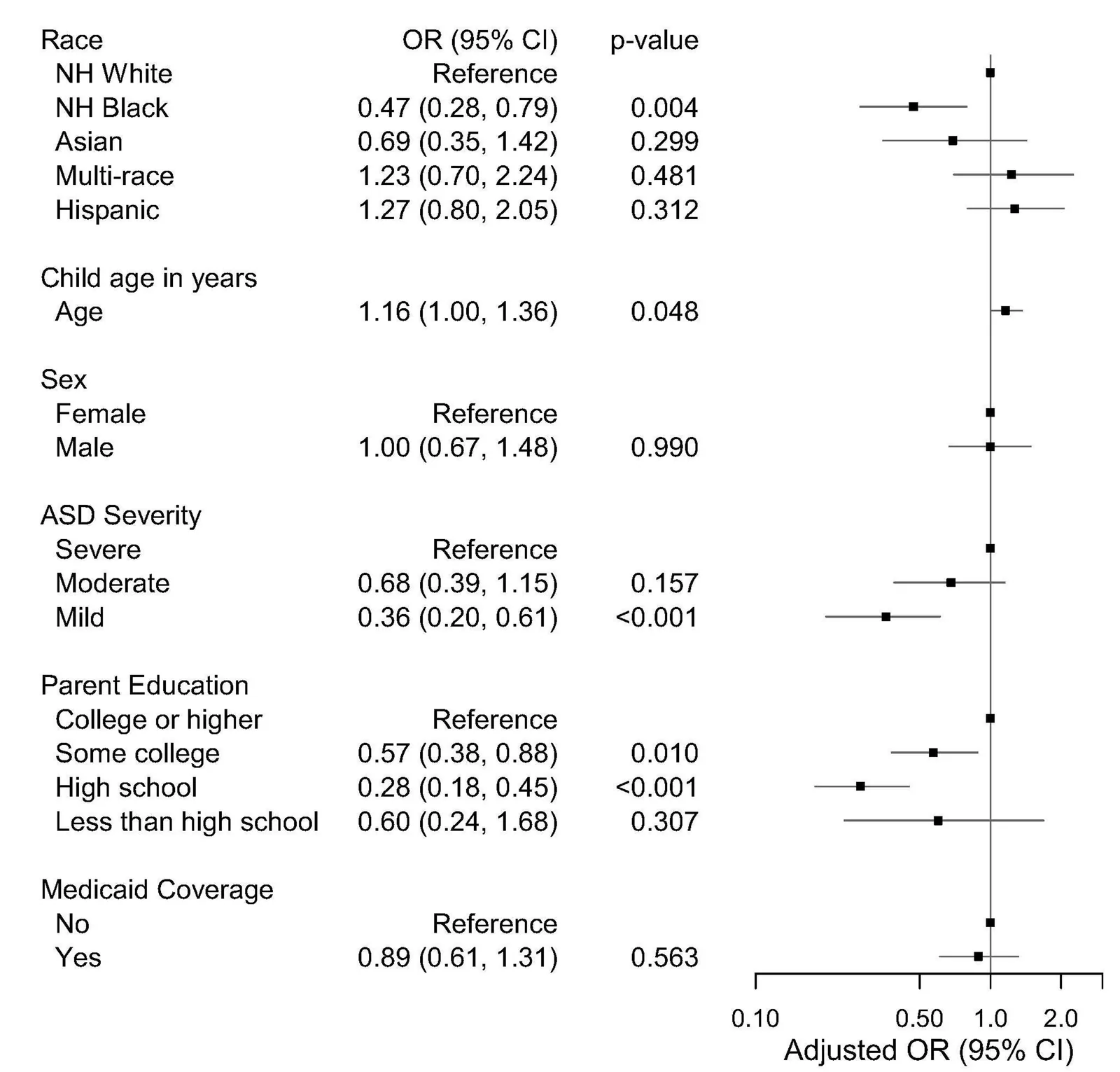
Adjusted ORs (95% CIs) of behavioral treatment among children with ASD. The figure shows the adjusted ORs of receipt of behavioral treatment from the multivariable logistic regression model. ASD, autism spectrum disorder; NH, non-Hispanic

## Discussion

Through analysis of the 2021-2022 NSCH data, we found that NH Black children with ASD were significantly less likely to receive treatment than NH White children. Disparities in caregiver-reported receipt of behavioral treatment were observed among NH Black children with ASD compared with NH White children, despite no statistically significant difference in age at diagnosis between these groups. These findings underscore the need for targeted efforts to ensure that NH Black children with ASD have equitable opportunities for developmental progress.

Over the past decade, racial disparities in the timing of ASD diagnosis between NH Black and NH White children with ASD have decreased [14]. However, a recent study demonstrated that Hispanic and NH Black children experienced the most pronounced disparities in access to ASD diagnostic resources and services compared with other racial and ethnic groups [15]. The present findings are consistent with recent literature on this topic. These findings suggest that racial disparities in caregiver-reported receipt of behavioral treatment warrant continued attention. While this study did not directly assess barriers to care, prior literature suggests that disparities in treatment receipt may reflect underlying inequities in access to behavioral health services [15].

Prior research has demonstrated that earlier behavioral intervention is critical for developmental outcomes and may reduce the severity of ASD symptoms in young children [10]. The US CDC recommends screening for ASD at 18-24 months of age and initiating behavioral therapy at younger ages to optimize developmental outcomes. Early intervention usually starts at two to three years of age [10]. However, our study found racial and ethnic differences in caregiver-reported receipt of behavioral treatment among children with ASD. Age at ASD diagnosis did not differ substantially across racial groups, with a range of 2.23 to 2.61 years. However, when analyzing the responses to the question above, “At any time during the past 12 months, did this child receive behavioral treatment for autism, ASD, Asperger’s disorder, or pervasive developmental disorder, such as training or an intervention that you or this child received to help with their behavior?”, a higher proportion of minority children with ASD reported not having received treatment compared with NH White children with ASD. Studies have reported financial burdens, discrimination, fear of stigma, lack of access to health care, and overall lack of knowledge about ASD as potential reasons for disparities in delayed ASD diagnosis [12,13]. These factors represent plausible explanations for disparities in treatment initiation among children with ASD, although they were not directly evaluated in the present study and warrant further investigation.

There are several limitations to this study. As this is a cross-sectional analysis, causal inferences between race and ASD treatment cannot be established. Because ASD diagnosis, treatment, and related characteristics were based on caregiver self-report, findings may be subject to recall bias or social desirability bias. Cases in which parents or guardians were unaware that their young child had ASD would not have been captured in our study. Study responders may be systematically different from nonresponders, and missing information may have affected the study results [16]. This study assessed only whether behavioral treatment was received, not the quality or intensity of the services provided. Only complete responses were included in the analysis; unanswered responses were excluded. However, a relatively low percentage of questionnaire responses were missing, and we used imputed data from the NSCH for race and ethnicity variables. Because analyses were conducted without applying NSCH survey weights or accounting for the complex survey design, the findings may not be nationally representative and should be interpreted as estimates of association within the study sample rather than population-level estimates. The study cannot rule out residual confounding. Unmeasured confounders, such as household income, geographic region, provider availability, or other social determinants of health, may also have influenced the observed associations. The relatively small sample sizes for some racial/ethnic groups, particularly NH Asian and NH Black children, may have reduced statistical precision and limited the ability to detect differences between groups. Findings for these subgroups should be interpreted with caution. Despite these limitations, this study has strengths. We used a large national sample of children with ASD, allowing examination of differences in caregiver-reported behavioral treatment across racial and ethnic groups among children younger than five years.

## Conclusions

Among a large national sample of US children with ASD, we observed a racial disparity in caregiver-reported receipt of behavioral treatment, with NH Black children less likely to receive treatment than NH White children despite similar ages at diagnosis. Given the cross-sectional design of this study, these findings cannot establish causality or determine whether treatment was delayed relative to diagnosis; rather, they indicate an association between race and reported treatment receipt at a single point in time. These findings highlight the importance of continued efforts to promote equitable receipt of behavioral treatment among children with ASD, particularly for NH Black children.
